# Spectroscopic Insight into the Role of Surface Oxygen Vacancies in the Detection of NO_2_ in SnO_2_‑Based Chemoresistive Gas Sensors

**DOI:** 10.1021/acssensors.5c04098

**Published:** 2025-12-13

**Authors:** Stefan Kucharski, Michael Vorochta, Lesia Piliai, Andrew M. Beale, Christopher Blackman

**Affiliations:** a Department of Chemistry, 4919 University College London, London WC1H 0AJ, U.K.; b Research Complex at Harwell, Rutherford Appleton Laboratory, Didcot OX11 0FA, U.K.; c Department of Surface and Plasma Science, Faculty of Mathematics and Physics, 37740 Charles University, Prague 8 180 00, Czechia

**Keywords:** conductometric, NAP-XPS, resistance, sensor, spectroscopy, vacancy

## Abstract

Despite widespread use of SnO_2_-based conductometric gas sensors, the sensing of NO_2_ remains poorly described on the atomic scale, limiting the design of next-generation sensors. Here, near-ambient-pressure X-ray photoelectron spectroscopy combined with in situ resistance measurement was used to investigate the interaction between NO_2_ and a SnO_2_-based sensor at room temperature and 300 °C. Through stepwise exposure and evacuation cycles, we tracked real-time changes in the O/Sn atomic ratio and electronic structure alongside the macroscopic resistance response. Exposure to NO_2_ consistently increased the O/Sn ratio, indicating the healing of surface oxygen vacancies, and this effect directly correlated with an increase in resistance. At room temperature, the response was cumulative and irreversible, while at high temperatures, it was rapid, reversible, and saturated at lower gas concentrations. These findings directly support vacancy-modulated “surface conductivity” and provide experimental validation that NO_2_ sensing in SnO_2_ occurs via modulation of shallow donor concentrations, rather than through the classical description of ionosorption of charged oxygen species. The results contribute to an emerging unified model of gas sensing and offer insight into how dynamic equilibrium between vacancy healing and regeneration underpins temperature-dependent sensor behavior.

## Graphical abstract

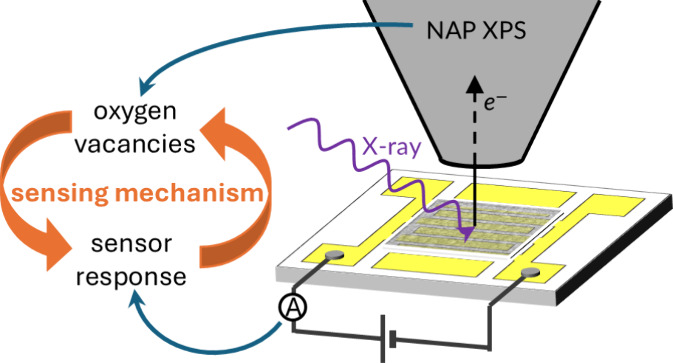

Since their introduction in the 1970s, conductometric gas sensors (CGS) have provided an inexpensive and easily deployable method for monitoring the concentrations of harmful gases. Among the most widely used CGS materials is SnO_2_, which is often employed for detection of “reducing” gases, for instance CO and volatile organic compounds. However, SnO_2_ also shows excellent sensitivity toward NO_2_,^1^ a known greenhouse gas and carcinogen emitted primarily from combustion processes. Considering that much of the NO_2_ emissions come from disperse sources and that dangerous annual average exposure levels of NO_2_ are as low as 53 ppb,^1^ there is an ever-increasing need for small, inexpensive NO_2_ sensors; this niche could be filled by SnO_2_-based CGS, which have been shown to detect NO_2_ levels as low as 5 ppb even at room temperature.^2^ However, despite the excellent sensitivity of SnO_2_ to NO_2_, the sensors still suffer from undesirable characteristics including long recovery times and interference from humidity.^3^ In order to address these shortcomings, it is essential to have greater atomistic insight into the surface chemistry governing gas sensor response in SnO_2_, to allow a design-led approach to new sensor development.

During the past several decades, the sensing mechanism of SnO_2_ has predominantly been explained in terms of the so-called “ionosorption” model, which involves formation of charged oxygen adsorbates on the surface of SnO_2_, resulting in an electron depletion layer under the surface, effectively increasing the resistance of a particulate sensitive layer in the presence of oxygen (for an *n*-type material).^4,5^ Despite extensive research, there is no experimental evidence for the monatomic oxygen adsorbates underlying “ionosorption”,^6^ and the model does not account for other effects observed in CGS, such as sensor drift—the change in sensor’s baseline resistance over extended periods of time. More recently, a model describing the sensitive behavior of SnO_2_ in terms of near-surface oxygen vacancies (V_O_) has emerged, supported by a large body of both empirical and computational studies.^7–9^ The numerous pieces of evidence in support of this description, herein referred to as the “surface conductivity” model, have recently been summarized here.^10^ In this description, the presence of (near-)­surface V_O_ acts as shallow electron donors,^11^ forming a conductive two-dimensional gas of delocalized electrons.^12^ In this case, the sensor’s resistance becomes a function of the equilibrium between the formation of V_O_ via reactions with reducing gases (or spontaneous reduction) and V_O_ healing via reactions with oxygen and oxidizing gases, such as NO_2_.

The advent of operando spectroscopy has provided a gateway into the observation of the dynamics of SnO_2_ surface chemistry under target gas exposure from joint micro- and macroscopic points of view. Some notable examples are IR and UV–vis diffuse reflectance spectroscopy,^7,13,14^ X-ray absorption spectroscopy,^15^ and near-ambient-pressure X-ray photoelectron spectroscopy (NAP-XPS) experiments.^16–19^ Such operando studies clearly show lattice oxygen removal, i.e., V_O_ formation, in SnO_2_ under temperatures and atmospheres relevant to the detection of reducing gases such as CO, H_2_, or ethanol.^7,15,17–19^ Moreover, some of these studies, including our own operando NAP-XPS experiments,^17,18^ feature concurrent resistance measurements that show a clear correlation between the concentration of V_O_ and the measured resistance, indicating that sensor response is intimately linked to near-surface stoichiometry of SnO_2_. In light of the insight gained from these investigations into the effect of oxygen^17^ and the effect of the reducing gas CO^18^ on SnO_2_-based CGS, we have applied the same NAP-XPS methodology to investigate the surface chemistry and corresponding resistance change upon exposure to the oxidizing gas NO_2_, which is the subject of this work.

## Experimental Section

The experiments presented herein were performed on a sample SnO_2_-based conductometric sensor with interdigitated electrodes. The details of the sensor’s design and manufacturing have been previously published elsewhere.^17^ The same sensor was used in both experiments described below. The test gas was prepared by mixing 20 cm^3^ of NO_2_ gas (synthesized at Charles University, Prague) with argon (Linde, N6.0) up to a total volume of 2000 cm^3^, i.e., notionally 10,000 ppm NO_2_ (in argon). While this is significantly higher than the typical NO_2_ detection range, the gas is used in the experiments at 0.5 and 1 mbar pressures; therefore, the sensor is subjected to partial pressures of NO_2_ that correspond to theoretical atmospheric concentrations of 5 and 10 ppm, respectively.

The operando XPS measurements were carried out at Charles University, Prague, using a SPECS NAP-XPS system equipped with a Phoibos 150 hemispherical analyzer, a DeviSim NAP reactor cell, and a monochromated Al Kα X-ray source (1486.7 eV). The sample sensor was mounted on a sample holder with two electrical contacts for the onboard thermocouple and placed inside the NAP cell on a stage heated from below via cathode rays. The sensor’s electrodes were connected to the thermocouple terminals; due to that, the temperature readouts were obtained from the auxiliary thermocouple located within the NAP cell, which measures the stage temperature and is correlated with the sample temperature. The in situ resistance measurements were collected using a Keithley 6517B source meter set to source a 100 mV voltage, recording the resulting current at 1 s intervals.

The sensor was used to conduct two consecutive experiments, the first at room temperature (RT) and the latter at 300 °C (HT, high temperature). Prior to the first experiment, the sensor was “cleaned” in situ by heating in 5 mbar O_2_ to 500 °C for 5 h followed by heating for 1 h in UHV at the same temperature. Subsequently, the sensor was cooled in UHV to room temperature, at which point the RT experiment commenced. After the RT experiment was completed, the sensor was heated to 400 °C in UHV for 15 min to regenerate the surface and later cooled in UHV to 300 °C in preparation for experiment HT. Both experiments followed the same progression of steps, starting in UHV (step “before”) through 0.5 and 1 mbar of test gas (steps 1 and 2) back to UHV (step “after”) and finally again to 0.5 mbar (step “reintro”). At every step, the same set of XP spectra was collected, which includes a survey scan followed by high-resolution scans of the Sn 3d, O 1s, C 1s, and N 1s regions.

The spectra were analyzed in CasaXPS.^20^ The Sn 3d peaks were fitted with two Lorentzian asymmetric line shape (LF) components, one for each peak of the 3d doublet. The line shape parameters of the two components were adjusted to fit the spectra taken immediately before the first oxygen exposure (“UHV before”) and remained unchanged within each experiment. No constraints were imposed on the peak position, area, or full width at half-maximum (fwhm).

The O 1s peak was fitted with two components: the lattice peak, which corresponds to the oxygen contained within the SnO_2_ lattice, and “O third”, corresponding to all other oxygen present within the analyzed volume. The “O lattice” component was modeled with an LF line shape whose parameters were adjusted to match the calibration data collected on the same sensor after reduction and dehydroxylation were performed to achieve a clean surface and before oxygen exposure; at that point, the sample is notionally free of adsorbates and hydroxyls. Any changes to the shape of subsequent photoemission measurements are modeled by the “O third” component, which uses a symmetrical Lorentzian line shape (LA). All parameters remained unconstrained, including those of the “O third” component.

The C 1s region was fitted with four Gaussian–Lorentzian (GL) components corresponding to hydrocarbons (C–H), alcohols (C–OH), ketones (C=O), and esters (O–C=O). The fwhm of C–OH, C=O, and O–C=O were constrained to be the same as the fwhm of the C–H peak, and their BE positions were constrained to, respectively, 1.5, 2.5, and 4.0 eV above the C–H peak. These scans were fitted using a procedure presented by McIntyre et al.^21^ that allows estimation of the amount of oxygen associated with the organic contaminants as opposed to those originating from the SnO_2_ (further discussion of O calc and the calculated values are available in the SI).

The O/Sn and “O third”/Sn ratios were calculated by dividing the areas of the O components by the sum of the two components of the Sn 3d emission normalized using RSF values (2.93 for O 1s and 25.1 for Sn 3d). The O/Sn ratio was also calculated for the set of spectra collected on an “as-received” sensor, prior to any thermal treatment or gas exposure, to provide a point of reference for the reduction state of the sensor’s surface following thermal treatments under the low-pressure conditions of NAP-XPS; the value obtained was 1.29. The amount of organic oxygen estimated to be contributed to the O 1s spectra from that carbon overlayer (“O calc”/Sn) ranges between 0.03 and 0.11 and accounts for 15–80% of the “O third peak” depending on the step (the detailed table of atomic ratios obtained in this experiment is presented in the SI). Therefore, the trends in the intensities of “O third” and the oxygen-containing C peaks do not match, which is indirect evidence of “O third” originating from the only other likely candidates (given the absence of other peaks in the spectrum), i.e., oxygen adsorbates, hydroxyls, or possibly defective surface lattice atoms.

## Results and Discussion

### Spectral Analysis

As an example of the collected data, two sets of spectra are presented in Figure 1, one each from the RT and HT experiments. The remaining spectra collected in both the RT and HT experiments are presented in the SI. The same analysis, which is described below, was applied to each set of spectra and used as the basis of the consistent quantification of the XPS data.

**Figure 1:**
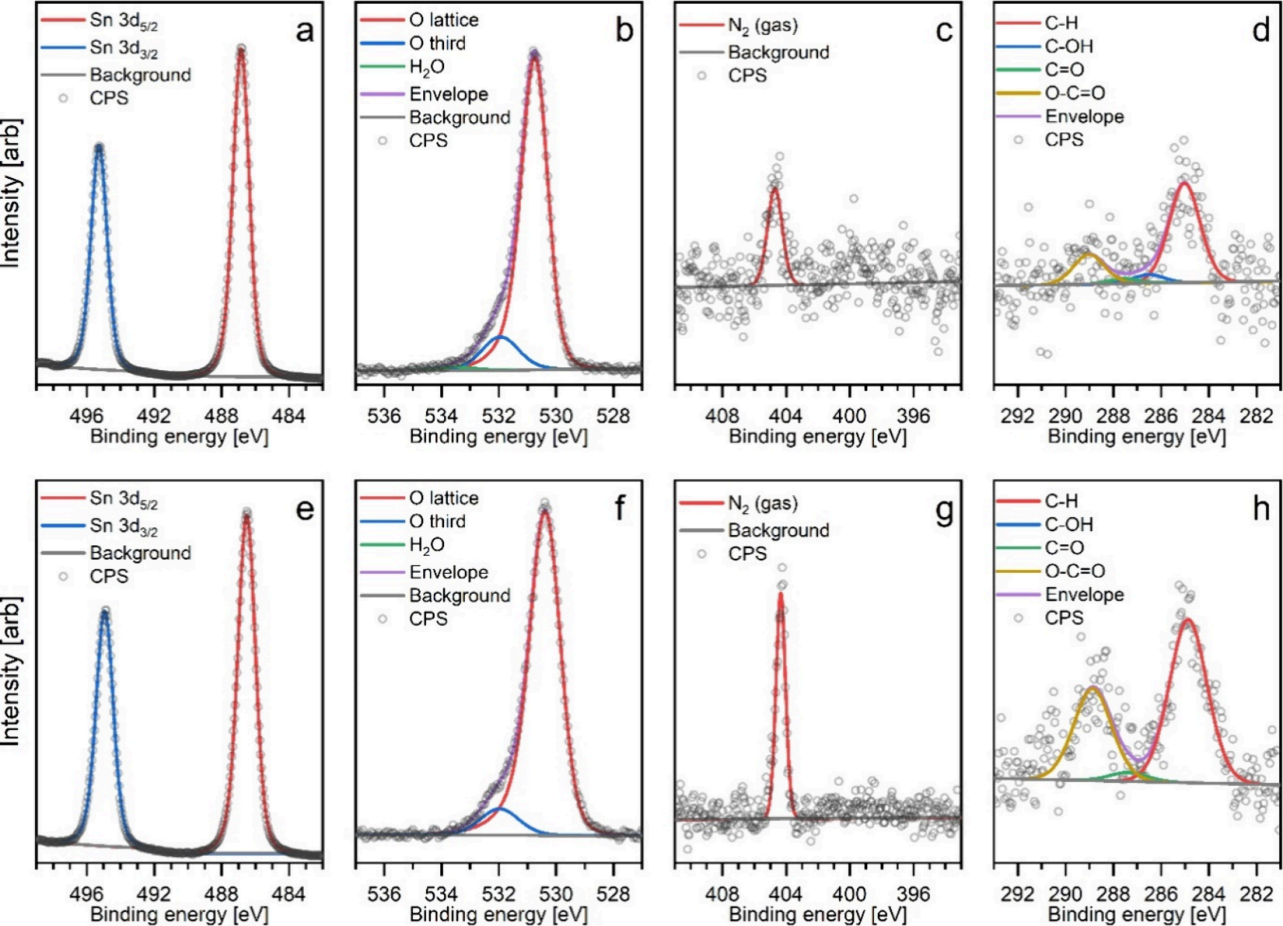
Two sets of XPS spectra collected during step 2 (1 mbar of test gas) during experiments RT (a–d) and HT (e–h). Each set of spectra consists of Sn 3d (a, e), O 1s (b, f), N 1s (c, g) and C 1s (d, h) high-resolution scans.

The Sn 3d region spectra were fitted with two peaks corresponding to the 5/2 and 3/2 emissions of the 3d doublet of the Sn^4+^ chemical state. Although, as outlined further below, there is evidence of substoichiometry of the SnO_2_ lattice in the relative intensity of the Sn 3d and O 1s peaks in these experiments, Sn^2+^ is not distinguishable from Sn^4+^ in the Sn 3d spectra of SnO_2_, as both species produce emission at the same binding energy.^22^ The shifts in the binding energy position of the Sn 3d_5/2_ peak between the steps of these experiments were used as indicators of band bending, as the binding energy scale is referenced to the Fermi energy within the analyzed volume; the validity of this approach was confirmed by comparing the shifts with those also observed in the collected O 1s spectra, which showed close agreement, indicating a relative change in Fermi energy as opposed to a change in the chemical environment (oxidation state).

In the O 1s region, several different species were observed; the main emission comes from the lattice oxygen within SnO_2_, denoted as the “O lattice”. The second peak, located at the higher binding energy side of the lattice peak, likely results from a variety of oxygen species including adsorbed oxygen, surface hydroxyls, or organic contaminants. Since the identity of this peak is uncertain and there are many possible sources not related to the SnO_2_ lattice, it was given the name “O third” for “third-party species’”. Additional discussion on the origin and characteristics of “O third” can be found further in this paper and in our previous publications.^17,18^

The N 1s region was not very informative, as the only reliably observed peak corresponded to N_2_, likely coming from contamination of the NO_2_/argon test gas with atmospheric air (80% N_2_) during its preparation. The contamination was significant, as the peak corresponding to molecular NO_2_ had a lower intensity than diatomic N_2_, and hence was not directly observed during the experiments due to the low partial pressure of the NO_2_ test gas mixture and overall low intensity of the N 1s spectra. However, an N 1s spectrum collected at 2 mbar of test gas pressure shows a clear signal corresponding to NO_2_ (see Figure S1 in the SI).

Finally, the C 1s region showed some carbonaceous contamination on the surface, which varied slightly throughout the experiments. The C 1s region was quantified using a procedure first published by McIntyre et al.,^21^ which allows a rough estimation of the amount of oxygen associated with the carbonaceous overlayer and hence the proportion of “O third” signal ascribed to these carbon-containing contaminants. The exact implementation of this method has been published previously,^17^ and its results are denoted as “O calc” in this paper.

### Experiment RT

The sensor’s resistance during experiment RT is presented alongside the temperature and test gas pressure in the NAP cell in the bottom panel of Figure 2. The baseline value of sensor’s resistance, which is used to calculate the sensor’s response, is taken at the end of step “before”, i.e., immediately before exposure to the test gas; in this experiment, that value was 1.5 kΩ, indicative of a partially reduced surface as expected after cooling from 500 °C in UHV.^23^ As soon as the test gas was introduced in step 1, the sensor’s resistance decreased initially, down to 400 Ω, which was likely caused by a variety of factors, including a sudden change in pressure, which typically causes resistance fluctuations, and “reducing-type” effects of H_2_O, which was present in the test gas as witnessed in its O 1s spectra (see Figure S1 in the SI). Following that initial drop, the resistance started to increase and reached 1.5 kΩ by the end of step 1, without showing signs of reaching a stable value (response (*R*) as resistance in test gas (Rg)/resistance (Ra); *R* = 1 when Ra is taken as resistance in step “before”). After the pressure was increased in step 2, the resistance continued to increase at a higher rate compared to step 1 and reached 170 kΩ (*R* = 113) just before the gas was evacuated, again without having reached a stabilized value. During the step “after”, the resistance continued increasing (slightly) until the test gas was completely evacuated (note the pressure dropping gradually around 17,000–18,000 s), at which point it reached a stable value of 240 kΩ (*R* = 160). Upon reintroducing the gas mixture during the final step, the resistance again decreased slightly at first (similar to step 1) and then started to increase until a stable value of 320 kΩ was reached (*R* = 213). Given that the effect of O_2_ on SnO_2_ sensors at room temperature is negligible^17^ and the other gases present in the mixture are either inert or expected to induce a reducing-type sensor response, NO_2_ must be responsible for the substantial resistance change (from 1.5 to 320 kΩ) observed in this experiment.

**Figure 2:**
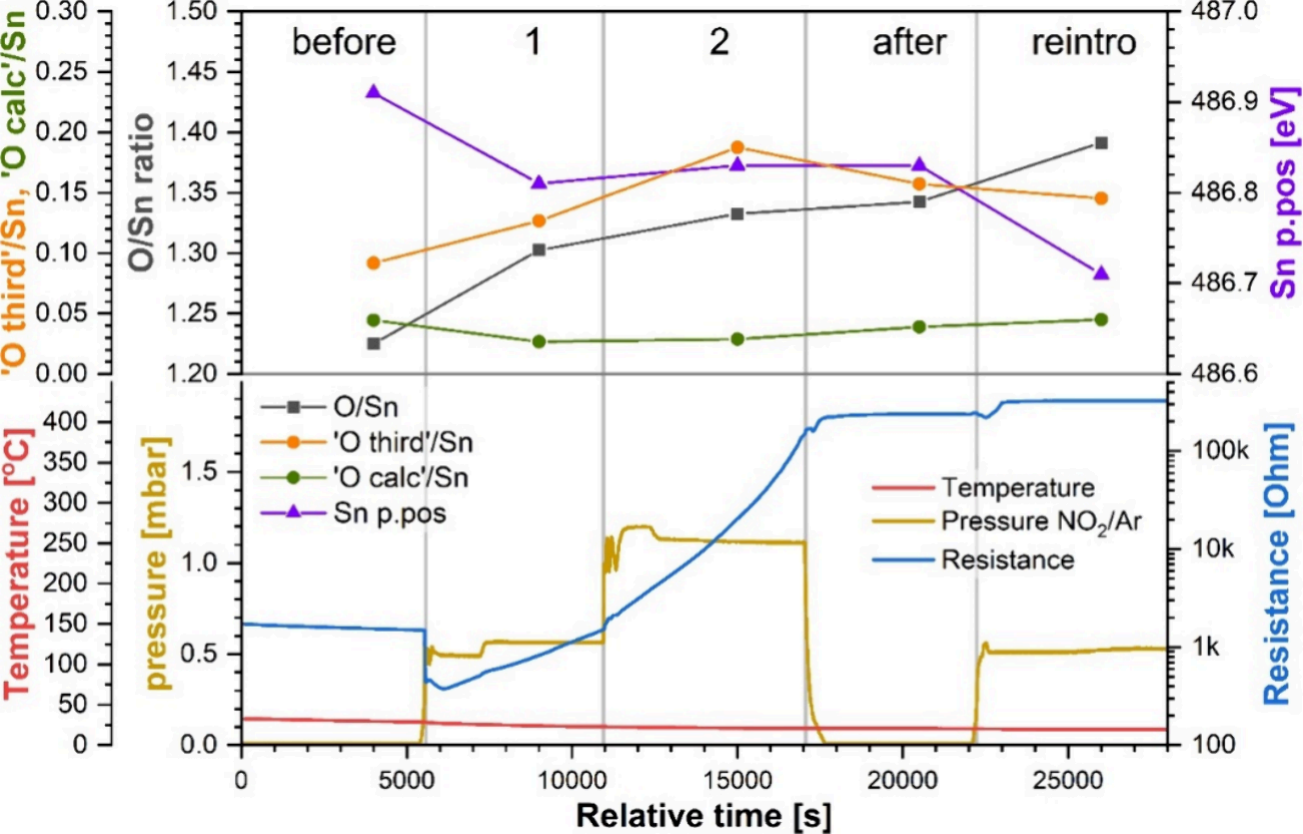
Compiled results of experiment RT. (Top panel) XP spectra quantification results: Sn p.pos: position of the Sn 3d_5/2_ peak; O/Sn: SnO_2_ stoichiometry; “O third”/Sn: amount of additional oxygen species on the surface; “O calc:/Sn: estimated amount of oxygen in the organic surface contaminants. (Bottom panel) Sensor’s resistance and temperature and pressure of the test gas in the NAP cell.

The quantitative analysis of the XP spectra, which complements the resistance measurements, is presented in the top panel of Figure 2. The first parameter therein, Sn p.pos., tracks the position of the Sn 3d_5/2_ peak, which indicates the changes in the Fermi level position near the surface, i.e., band bending. The other parameters reflect the changes in the relative abundance of oxygen species relative to the number of Sn atoms since the latter is considered invariant and therefore normalizes the observed intensities of O 1s emission. The primary of these, derived from the “O lattice” peak, is the O/Sn ratio, which is a measure of the stoichiometry of the SnO_2_ lattice. The further two parameters trace the amount of oxygen species besides SnO_2_, which may include oxygen adsorbates, hydroxyls, or organic contaminants (“O third”/Sn ratio) and the estimate of oxygen contained in the organic contaminants based on the C 1s spectra (“O calc”/Sn).

The band bending analysis shows that the position of the Sn 3d peak shifted from 486.9 to 486.8 eV upon the introduction of the test gas in step 1, indicating an upward band bending of 0.1 eV. The peak position did not change with a further increase in gas pressure or during the subsequent evacuation during step “after”, remaining at 486.8 eV. After reintroducing the gas mixture in the final step of the experiment, the bands bent upward again by about 0.1 eV, with the final Sn 3d peak position of 486.7 eV. The upward direction of band bending is indicative of an oxidizing (i.e., resistance-increasing) influence of the test gas on the surface, which is consistent with the effect of NO_2_ on SnO_2_, and the cumulative pattern of the changes (i.e., nonreversal in UHV) indicates that the changes are effectively permanent in this temperature regime.

The O/Sn ratio, which determines the stoichiometry of the surface, corresponds well to the trends observed in band bending. The initial value, at 1.23, was relatively low compared to the “as-received” value of 1.29, indicating an atypically reduced surface, which could potentially inhibit some sensing mechanism pathways, for example, the formation of nitrites. However, during the experiment, the ratio increases to 1.30 during the initial exposure to the test gas in step 1 and further to 1.33 in step 2. Following the evacuation of the test gas in step “after”, the O/Sn ratio remains largely unchanged at 1.34, and the subsequent reintroduction of the test gas increases it further up to 1.39. This final value is much higher than the “as-received” reference and so should represent a surface with a substantial relative density of surface lattice atoms. This range of O/Sn values is consistent with those we observed during reaction of SnO_2_ with CO,^18^ indicating that the reduction and oxidation of the sensor’s surface occur over a set O/Sn range, perhaps governed by the lattice oxygen mobility and the gas-surface dynamics under the experimental temperature and pressure. With an overall increase throughout the experiment and with two significant steps upon introducing the test gas in steps 1 and “reintro”, the trend in surface stoichiometry in this experiment is consistent with the trends in both band bending and resistance change.

The “O third”/Sn ratio, initially at around 0.1 during step “before”, also showed an increase in the first two steps (up to ca. 0.2), which may be attributed to the formation of surface hydroxyls (dissociative adsorption of H_2_O) or possibly NO_*x*_ adsorbates. However, no indication of the latter was observed in the N 1s spectra, so their contribution to the “O third” peak must also be negligible; XPS is only slightly more sensitive to oxygen than nitrogen, and hence, the absence of an N 1s peak related to NO_*x*_ species means that any accompanying O 1s peak is expected to at most be at the limit of detection. The “O third”/Sn ratio reached a maximum value of ca. 0.2 during step 2 and then decreased slightly during the gas evacuation, down to 0.16. Subsequent test gas reintroduction had little effect on the intensity of the “O third” component, which decreased ever so slightly to 0.15. Although there are organic contaminants present on the sensor’s surface in this experiment, as evidenced by the C 1s spectra, the “O calc”/Sn estimation suggests that the oxygen contained within accounts only for between a quarter and a half of “O third” emission; consequently, O_2_ adsorbates and H_2_O (surface hydroxyls) remain the only prominent contributors to this peak. As already established, both these gases result in the formation of an “O third”-type peak but they either have a reducing effect (H_2_O)^24^ or no effect on the sensor’s resistance at this temperature,^17^ so neither can account for the substantial resistance increase observed in this experiment. Considering the relative invariance of the intensity of “O third”, a lack of correlation between it and the resistance measurements, and the previously obtained evidence of the effects of O_2_ and H_2_O on SnO_2_ sensor resistance, it is unlikely that this peak holds information relevant to the sensing mechanism of SnO_2_.

Although no unambiguous determination regarding the various possible nitrogen oxide adsorbates can be made based on the spectra, since, if they are present, they are below the detection limit of the XPS experiment, the increase in surface stoichiometry of SnO_2_ (O­(lattice)/Sn ratio from 1.23 to 1.39) is certain and in line with expectations considering the large increase in resistance observed during this experiment (from 1.5 to 320 kΩ, *R* = 213). These results suggest that the dominant effect determining the sensor’s resistance (and therefore the response to NO_2_) is the increasing stoichiometry of the surface, i.e., the healing of oxygen vacancies, which act as shallow electron donors. Moreover, a pronounced increase in resistance was observed at room temperature in this experiment relative to that observed on exposure to oxygen.^17^ This suggests that NO_2_ can efficiently heal oxygen vacancies even at low temperatures, in contrast to O_2_ that requires elevated temperatures to dissociate (heal vacancies) at an observable rate.^25^ The substantial density of surface vacancy sites expected for SnO_2_-based gas sensors, even in a background of atmospheric O_2_ (20% O_2_), may explain the excellent sensitivity of SnO_2_ to NO_2_ at room temperature, which has been suggested previously in the literature.^26–31^

In summary, at room temperature, a sensor (resistance) response is observed on exposure of (partially spontaneously reduced) SnO_2_ to a NO_2_/argon mixture, in which the resistance change is correlated with a change in the concentration of surface oxygen vacancies (the O/Sn ratio). This key result is in stark contrast to the sensor’s exposure to O_2_ and CO under the same conditions, where little to no effect on the surface stoichiometry was observed (O_2_ adsorbs to the surface without a significant resistance change or dissociation,^17^ and CO fails to reduce the surface or lead to a resistance change^18^). This therefore identifies a possible approach to tuning the selectivity of the sensor’s response to NO_2_, where operating at room temperature would selectively detect NO_2_ without being affected significantly by any changes in the O_2_ background or presence of reducing CO. However, further experiments (such as codosing of the sensor with mixtures of these gases and validating the results in ambient conditions) would be necessary to confirm this hypothesis.

### Experiment HT

The resistance of the sample sensor during experiment HT (analogous to RT but performed at 300 °C instead of room temperature) is presented alongside the temperature and test gas pressure in the bottom panel of Figure 3. During step “before” the sensor’s resistance increased slightly as the temperature was decreasing toward a stable value and then started to decrease slightly toward the end of the step likely due to a slow spontaneous reduction of the sensor at this temperature,^25^ the final value before the gas introduction was 4 kΩ. Even though this experiment was performed at a higher temperature than RT and hence the sensor’s initial resistance might be expected to be lower, the sensor’s surface was more oxidized relative to the start of experiment RT (see discussion of the O/Sn ratio below), i.e., contained a lower concentration of oxygen vacancies (electron donors). The influence of the balance of these different effects on measured resistance is difficult to predict but here leads to a higher initial resistance than at RT.

**Figure 3:**
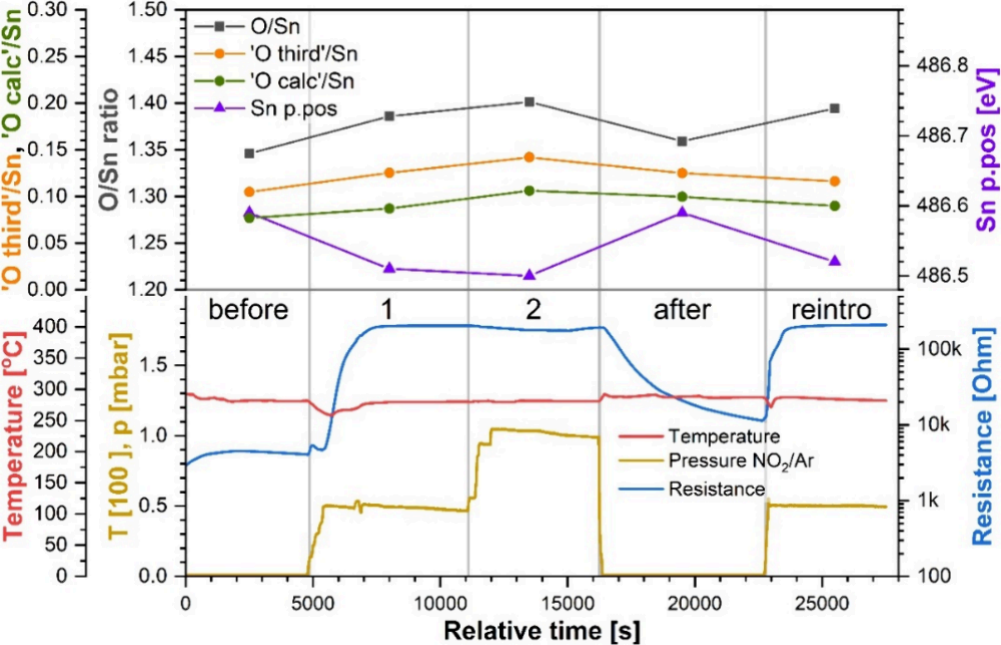
Compiled results of experiment HT. (Top panel) XP spectra quantification results: Sn p.pos: position of the Sn 3d_5/2_ peak; O/Sn: SnO_2_ stoichiometry; “O third”/Sn: amount of additional oxygen species on the surface; “O calc”/Sn: estimated amount of oxygen in the organic surface contaminants. (Bottom panel) Sensor’s resistance and temperature and pressure of the test gas in the NAP cell.

Introducing the test gas in step 1 resulted in a marked resistance increase, up to 200 kΩ (*R* = 50), with a much greater rate of resistance increase than that seen at RT, but no further change was observed upon increasing the pressure of the test gas in step 2. However, the subsequent evacuation to UHV caused the sensor’s resistance to gradually decrease to 11 kΩ (*R* = 3). Although this value is slightly higher than the initial 4 kΩ, the resistance did not reach a stable value before the end of step “after”; therefore, with the continued decrease, the initial resistance of 4 kΩ might have been recovered given sufficient time. After the test gas was reintroduced in the final step, the resistance increased again to a stable value of 210 kΩ (*R* = 52), very nearly the same value as that in step 1. The sensor’s return to nearly the initial value in step “after” and back to the same value in step “reintro” as in step 1 indicates not only complete recovery of the sensor but also good reproducibility of the sensor’s response under these conditions.

The quantification parameters of the XP spectra collected in this experiment are shown in the top panel of Figure 3. The initial position of the Sn 3d peak at 486.6 eV is lower than the corresponding value in experiment RT (486.9 eV); this shift to lower binding energy is typically observed with increasing temperature.^32^ After the test gas was introduced in step 1, the peak moved to 486.5 eV, which corresponds to an upward band bending of 0.1 eV; the further increase in pressure during step 2 had no effect on the position of the Sn 3d peak. However, the evacuation of the test gas in step “after” caused unbending of the bands, with the Sn 3d peak returning to its original position of 486.6 eV, and further reintroduction of the test gas in the step “reintro” resulted again in upward band bending of similar size, ca. 0.1 eV. Overall, the band bending shows good reversibility at this temperature, in agreement with the observed trends in resistance.

Completing the picture of correlated trends in this experiment are the changes in the surface stoichiometry of the sensor. Starting at an initial value of 1.35, the O/Sn ratio increased to 1.39 in step 1 and later only negligibly further increased to 1.40 in step 2, reflecting the significant initial change and no further change observed in resistance and band bending in these two steps. Furthermore, the O/Sn ratio returned to 1.36 in step “after” when the test gas was evacuated, nearly recovering the initial value, and then increased again to 1.39 in step “reintro”, which is identical to that in the equivalent test gas pressure in step 1. These changes in sensor’s stoichiometry are correlated with not only the observed band bending but also the changes in sensor resistance. As the O/Sn ratio increased from 1.35 to 1.40, due to the presence of NO_2_ in the sensor’s atmosphere, the resistance increased from 4 to 200 kΩ (*R* = 50). Conversely, the decrease in resistance down to 11 kΩ (*R* = 3) in step “after” was accompanied by a decrease in the surface stoichiometry down to 1.36.

While the correlation between the sensor’s surface stoichiometry, band bending, and macroscopic resistance presents a compelling picture of sensor operation, no nitrogen-based adsorbates were observed in these experiments. Although we acknowledge that the detection limit of XPS, at ca. 1% relative abundance, is significantly larger than the expected density of charged surface adsorbates (which is determined by the Weisz limit),^6,33^ such as those commonly used to describe the sensor’s operation within the framework of “ionosorption” theory, the changes in surface stoichiometry are undeniable and their effect on surface conductivity is well-documented in the literature.^14,23,34^

In summary, at a high temperature (300 °C), a rapid initial sensor (resistance) response is recorded on exposure of SnO_2_ to a NO_2_/argon mixture, in which the resistance change is again correlated with a change in the concentration of surface oxygen vacancies; however, no further resistance change is recorded on increasing the concentration of the test gas. At high temperature, the response to NO_2_ is dynamic, recovering to (essentially) the initial resistance value on exposure to UHV.

## Results Summary and Discussion

A direct comparison of the two experiments provides a broader perspective of the meaning of the collected data. Starting with similarities, both experiments showed that the surface stoichiometry and band bending are closely correlated in their response to the test gas, showing a surface becoming oxidized on a microscopic scale, with an increasing O/Sn ratio (fewer V_O_) and a decreasing Fermi level (higher resistivity). Furthermore, the simultaneously measured sensor’s response also showed close correlation with the microscopic parameters, with a substantial resistance increase upon exposure to NO_2_. Together, these relationships show a complete micro- and macroscopic view of the direct link between the near-surface stoichiometry and the sensor’s response, providing another case study^17,18^ in favor of the vacancy-mediated “surface conductivity” model.^10^

As for the differences, the increase in the temperature in HT caused a significant change in how the resistance increased upon exposure to NO_2_. Unlike in the RT experiment, where the resistance increased steadily over extended periods after increasing gas pressure (NO_2_ concentration) and in a cumulative fashion (without recovery in UHV) up to a relatively high response (*R* = 213), in the HT experiment, the resistance increased much faster (reaching saturation within the time frame of each step) and reverted back to the original value when NO_2_ was removed, and the saturated response was much lower than in RT (cf. *R* = 50 (HT) vs 210 (RT)). These differences suggest that the temperature increase activates a reaction competing with the surface oxidation by NO_2_; possible candidates include desorption of the NO_2_ adsorbates, assuming a more traditional “ionosorption” model, or dynamic formation of oxygen vacancies under heating. In terms of the findings of this study, the latter is a clear favorite as the surface stoichiometry decreases upon exposure to UHV, i.e., oxygen vacancies are formed by spontaneous surface reduction, which reverses the oxidation of the surface induced by NO_2_ exposure. Nevertheless, we note that charged “ionosorbed” species could not be observed via XPS due to the exceedingly small surface densities described by the Weisz limit,^6,33^ and therefore, their involvement cannot be excluded based on these data.

Although the experiments presented here cannot directly exclude the involvement of “ionosorption”-type adsorbates, the literature may shed some light on the likely pathway of the atomistic interactions between the test gas and the surface. For example, Maiti et al. found NO_2_ and NO_3_^–^ species on the surface of SnO_2_, which disappeared upon heating to around 130 and 425 °C, respectively.^26^ However, a follow-up study determined that NO_3_^–^ species are predominantly formed on shaped particles with at least two nanoscaled physical dimensions;^35^ NO_3_^–^ species can be formed when two NO_2_ adsorbates, bound to the surface through one or both O atoms, “walk” randomly along the rows of surface Sn atoms by switching between monodentate and bridging bidentate geometries and come into proximity with one another,^26^ which happens more often when the Sn rows are short due to the nanoscale dimensions of the crystallites.^35^ Although the NO_3_^–^ species are less relevant to this work, in which unshaped micrometer-scale powder was used, it should be noted that they may play an important role in more realistic surface conditions, i.e., in the presence of water vapor and surface hydroxyls, as shown for In_2_O_3_.^36^ Therefore, the model system studied here likely represents one of the possible mechanistic pathways; further experiments involving codosing would be required to identify other pathways.

Given that experiment HT was carried out at 300 °C, the NO_2_ species should therefore have been quickly removed from the surface, which would manifest itself as quick stabilization of the resistance in step “after”. This does not match the observed trend in resistance, where a slow, gradual decrease was observed (NO_3_^–^, if present, would not be expected to desorb at 300 °C and hence would lead to an irreversible change in baseline resistance after evacuation, i.e., step “after”, which again was not observed). Consequently, neither direct NO_2_ adsorbates nor NO_3_^–^ species provide a consistent explanation for the observed data.

Further information on reactions taking place during sensing come from TPD studies, which show desorption peaks at similar temperatures to those reported by Maiti et al.^37–39^ However, these TPD studies also show that the desorption products contain significant quantities of NO,^37,38^ and, in one case, no NO_2_ at all.^39^ In fact, Tamaki et al. remarked in their paper when comparing TPD results after NO and NO_2_ exposure that the “desorption behavior was very similar” and that “the adsorbates starting from NO_2_ are essentially the same as those from NO”.^39^ Consequently, at least some of the adsorbed NO_2_ must bind to the surface permanently and break apart (to give NO) rather than desorbing intact (as NO_2_), i.e., NO_2_ effectively heals a surface oxygen vacancy. The spent adsorption sites (healed vacancies) can then be regenerated by spontaneous reduction of the surface; according to the TPD experiments of Tamaki et al., O_2_ starts to desorb at higher temperatures than NO_*x*_, only by around 400 °C. However, a measurable stoichiometry change of the SnO_2_ surface in UHV was reported by Semancik at al. at temperatures as low as 130 °C^23^ and by the current authors at 300 °C.^17^ Moreover, the O_2_ peak observed by Tamaki et al. was much larger than the NO peak (after NO_2_ exposure) and also present after NO exposure, indicating that it includes not only the oxygen deposited on the surface by NO_2_ dissociation but also the intrinsic oxygen of the SnO_2_ lattice. Therefore, the dissociative adsorption of NO_2_ and the spontaneous reduction of the surface are expected to play an important role in the sensor’s resistance change (according to the “surface conductivity” model of ionized oxygen vacancies acting as electron donors), with the former explaining the surface stoichiometry increase observed in this study and the latter explaining why the sensor’s behavior is reversible only above 130 °C (i.e., in the HT experiment, at 300 °C).

Looking further into the literature, DFT studies show that NO_2_ adsorption onto V_O_ in SnO_2_ results in vacancy healing and NO production,^26,40^ as shown in Figure 4; Figure 4a shows a (highly reduced) SnO_2_ surface featuring both in-plane and bridging oxygen defects (V_O_) interacting with a NO_2_ molecule in the ambient, during which an oxygen atom from the NO_2_ heals the bridging oxygen vacancy and NO is released (Figure 4b shows an identical interaction with a less reduced SnO_2_ surface, featuring only the bridging oxygen vacancy). As discussed earlier, the presence of surface V_O_ acts as shallow electron donors^11^ decreasing sensor resistance, and hence, the healing of the oxygen vacancies by dissociation of NO_2_ as shown in these models would decrease the V_O_ concentration and resistance would be expected to increase. This vacancy healing process by NO_2_ has been shown to be energetically favorable and subject to only a small activation barrier,^41^ indicating that it could occur readily even at room temperature. These findings are consistent with our results at room temperature in which both the sensor resistance and the V_O_ ratio increase (i.e., V_O_ decreases) on exposure to NO_2_. This behavior has also been observed elsewhere, with SnO_2_ shown to activate NO_2_ and selectively reduce it to NO and N_2_, with some studies reporting a conversion factor of over 90% at 550 °C.^42^ However, for SnO_2_ to act as a catalyst, it must also regenerate oxygen vacancies after reducing a NO_2_ molecule; otherwise, it would rapidly become “poisoned”, which is where its ability to spontaneously reduce comes into play. It is well-established that SnO_2_ reduces at elevated temperatures with the process starting at temperatures as low as 130 °C, which can lead to a significantly reduced surface at 500 °C.^18,25^ At the temperature of the HT experiment (300 °C), our SnO_2_ sensor is expected to regenerate oxygen vacancies due to this spontaneous reduction, and hence, unlike the RT experiment, the sensor resistance for HT returns to its original value during step “after”, while at RT, the sensor can only record the cumulative effect of NO_2_ exposure (because the temperature is insufficient to regenerate the previously healed oxygen vacancies). This would also explain why the recorded sensor response is larger at the lower temperature; the spontaneous reduction of the SnO_2_ that generates oxygen vacancies, which is a thermally activated process that can only occur at elevated temperatures, is no longer in dynamic equilibrium with the oxygen vacancy healing effects of the surface reaction with NO_2_, which can occur at room temperature, and hence, the vacancy healing of NO_2_ is dominant and a greater resistance change is observed overall.

**Figure 4:**
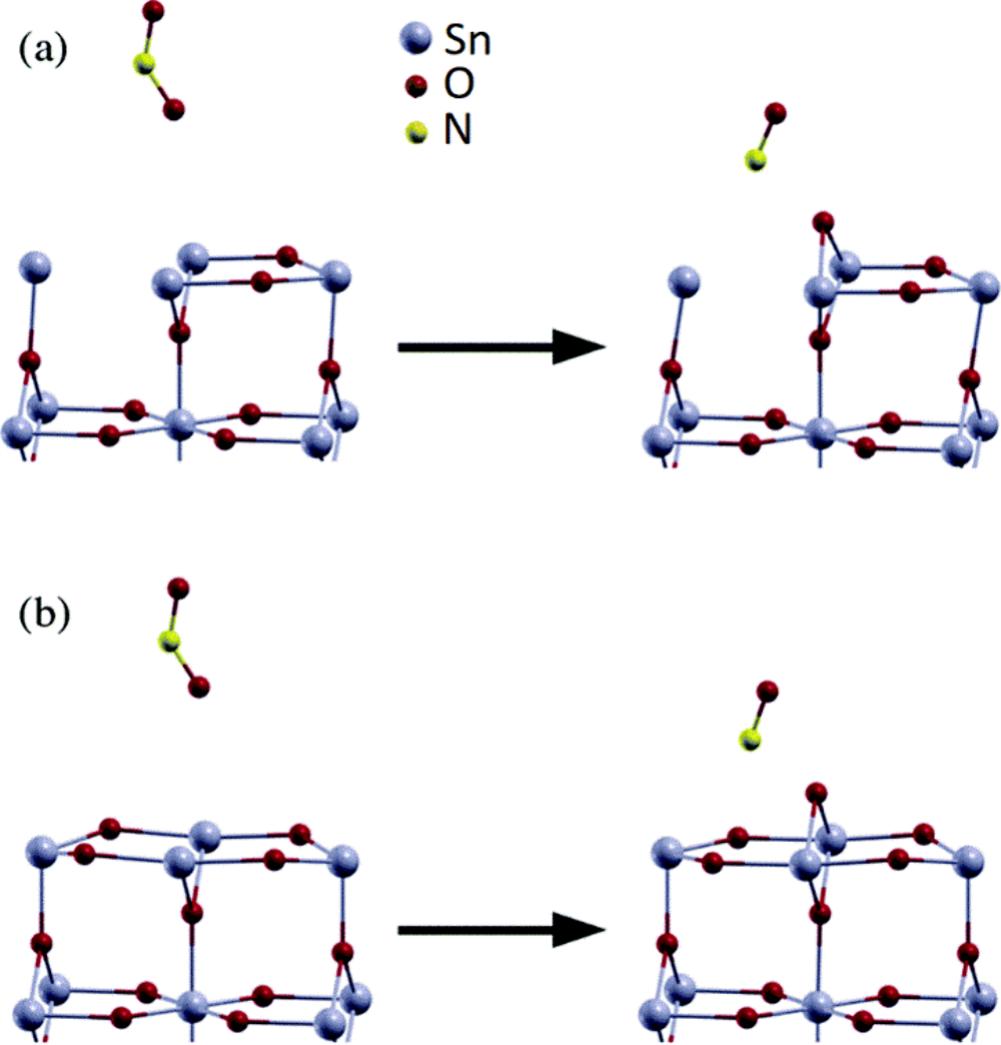
NO_2_ adsorption onto a reduced SnO_2_(110) surface with both in-plane and bridging oxygen vacancies (a) and only bridging oxygen vacancies (b). The NO_2_ molecule adsorbs with one of its oxygen atoms onto a bridging oxygen vacancy and dissociates to heal the surface, forming a weakly adsorbed NO molecule in the process. Color scheme: blue, Sn; red, O; yellow, N. Reproduced with permission from ref 40. Copyright 2016 Royal Society of Chemistry.

The conclusions drawn from the literature support the results of this experiment, where the NO_2_ oxidizes the surface by interacting with it and results in an observably larger O/Sn stoichiometry in the surface. This, in turn, results in a depletion of charge carriers and consequently increases the sensor’s resistance, producing a measurable sensor response. This description features prominently in the literature, supported by numerous empirical studies, and consequently, the gas-sensitive phenomena of the interaction between NO_2_ and SnO_2_ can be fully explained in terms of the dissociation of NO_2_ and healing of SnO_2_ surface oxygen vacancies. We note that this is unlikely to represent a full account of all the mechanisms governing the detection of NO_2_ on SnO_2_, which have previously been demonstrated to change depending on the sensor operating temperature, for example, in In_2_O_3_ between 130 and 350 °C.^36^

## Conclusions

This work presents two experiments investigating the effects of NO_2_ adsorption onto SnO_2_-based gas sensors at room and elevated (300 °C) temperatures. In both cases, the sensor’s resistance increased upon the introduction of NO_2_, indicating an oxidizing-type response, as expected for NO_2_. At the same time, the NO_2_ exposure leads to an observable increase in the O/Sn stoichiometry of the sensor’s surface, indicating the importance of oxygen vacancies in the gas sensing of SnO_2_.

The differences in the sensor’s behavior at room and elevated temperatures confirm which processes are important in response elicitation. At room temperature, the increase in resistance was gradual and cumulative, whereas at high temperatures, the increase in resistance was more instantaneous, reaching a stable value soon after pressure change and reversible in UHV, indicating that the processes responsible for response elicitation occur more readily at elevated temperatures. These observations are consistent with the spontaneous reduction of SnO_2_, which should not be a factor at room temperature (hence no response reversal in UHV) but causes the surface to return to the original state at 300 °C in the absence of NO_2_. Considering that the dissociation of NO_2_ on an oxygen vacancy is exothermic and only subject to a small activation energy,^41^ it is not surprising that response is observed even at room temperature, but equilibrium resistance is reached much faster at elevated temperatures.

The results of this work support the “surface conductivity” model of gas sensing on SnO_2_,^10^ where the sensor's resistance is determined by the density of near-surface oxygen vacancies acting as shallow electron donors and the sensor’s response is determined by how the target gas, in this case NO_2_, affects the equilibrium density of those near-surface vacancies. These findings are also consistent with our previous work on the adsorption of O_2_ and CO on SnO_2_,^17,18^ together presenting an ever more compelling picture of the importance of oxygen vacancies in the sensing of SnO_2_. However, we tension this by noting the inevitable “pressure gap” between experimentally accessible conditions in NAP-XPS and real-world application and also that the target gas detection typically occurs in a mixture of gases, some of which may affect the sensor or interact with the target gas. To prove the importance of oxygen vacancies in the sensing mechanism of SnO_2_, more studies should be conducted using an ambient containing a realistic concentration of O_2_, ideally coupled with ex situ gas sensing measurements of the same device under true atmospheric conditions to explicitly identify similar vacancy-mediated reactions responsible for the response of SnO_2_ to NO_2_.

## Supplementary Material



## Notes

Additional experimental details, procedure used for spectra fitting and quantification, and complete set of spectra collected in the experiments (PDF)

## Abbreviations

CGS: conductometric gas sensor
NAP: near-ambient-pressure
XPS: X-ray photoelectron spectroscopy
