# Supplementary material for: Spectroscopic Insight into the Role of Surface Oxygen Vacancies in the Detection of NO2 in SnO2‑Based Chemoresistive Gas Sensors
Source: ACS Sens. 2025 Dec 13;11(1):747–55. doi: 10.1021/acssensors.5c04098 (PMC12836335; doi:10.1021/acssensors.5c04098)
Supplement: Supplementary file 1 [file se5c04098_si_001.pdf]

Supporting Information

Spectroscopic insight into the role of surface oxygen vacancies in the detection of NO_2 in SnO_2-based chemoresistive gas sensors

Stefan Kucharski[a,b], Michael Vorochta[c], Lesia Piliai[c], Andrew M. Beale[a,b] and Christopher Blackman[a]*

[a] Department of Chemistry, University College London, London WC1H 0AJ, U.K.;

[b] Research Complex at Harwell, Rutherford Appleton Laboratory, Didcot OX11 0FA, U.K.;

[c] Department of Surface and Plasma Science, Faculty of Mathematics and Physics, Charles University, Prague 8 180 00, Czechia

Contents

Test gas spectra 2
RT Experiment Spectra 3
HT Experiment Spectra 4
Materials and Sensor Preparation 5
O calc estimation from C 1s spectra 7
Summary of the quantitative analysis – O/Sn, O third/Sn and O calc/Sn 8
References: 8

Test gas spectra

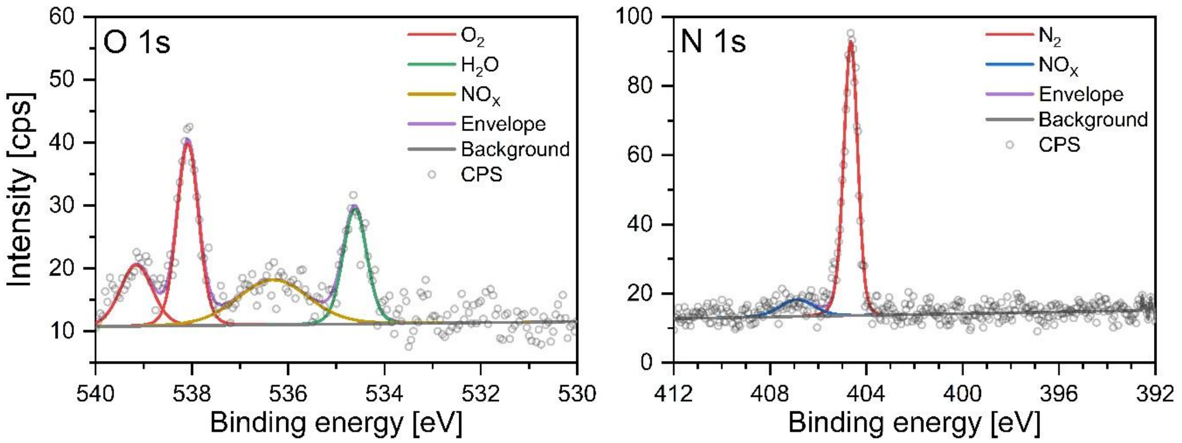

Figure S1: XP spectra of the O 1s and N 1s region of the test gas used for investigation. The sample was retracted away from the analysis site, leaving only the peaks corresponding to the gas phase. The splitting of the O 1s peak corresponding to the molecular oxygen originates from the final-state effects related to the spin-orbit coupling of the unpaired electrons in the antibonding orbitals of an oxygen molecule. Given the large FWHM of the peak denoted NO_X, it is likely a composite peak originating from a mixture of various nitrogen oxides, indicating that some of the NO_2 may have decomposed.

RT Experiment Spectra

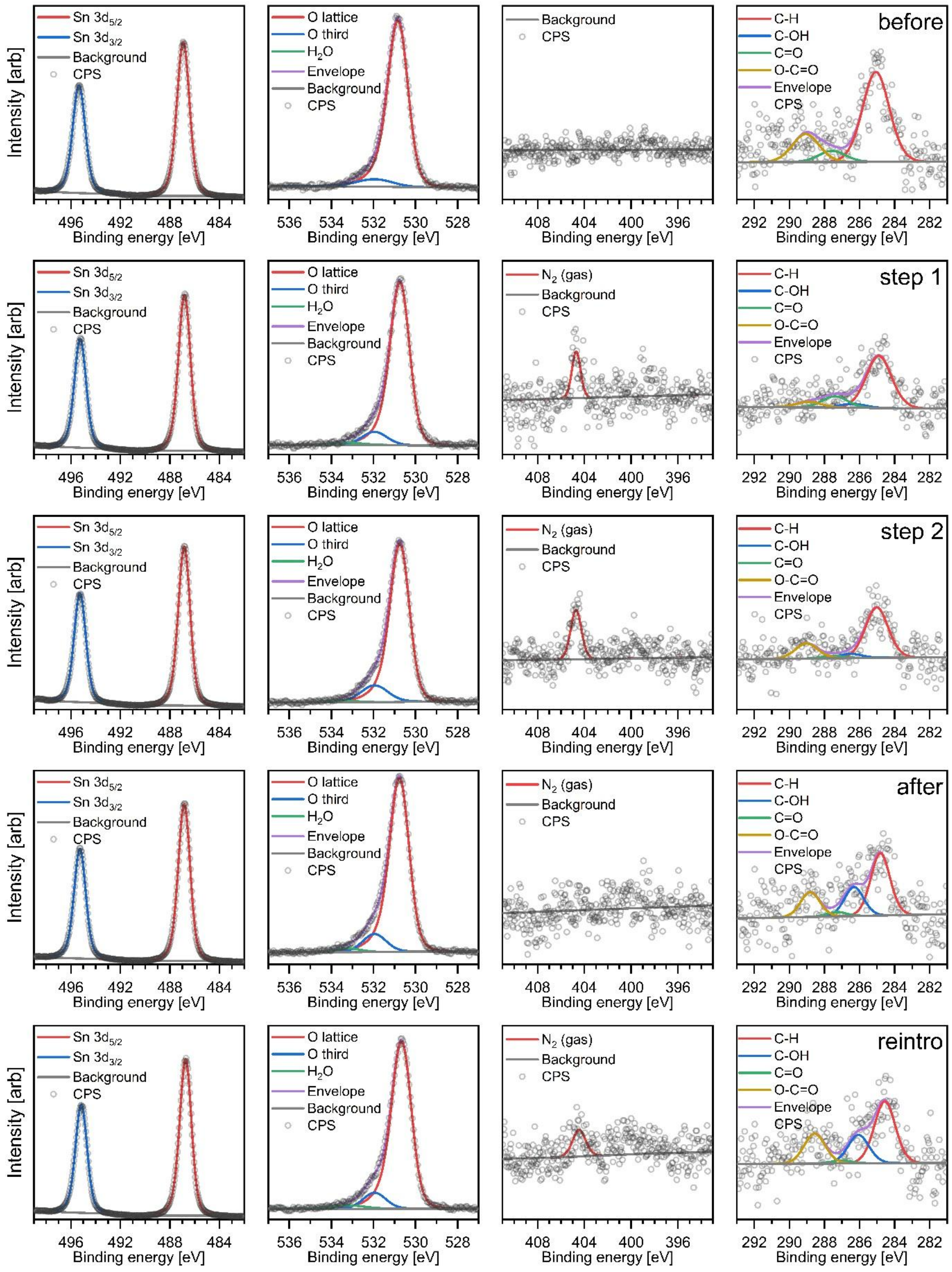

Figure S2: XP spectra of the Sn 3d, O 1s, N 1s and C 1s regions collected during the experiment RT. The label in the upper right corner of the C !s spectrum in each row corresponds to the experiment step during which that row was collected. The test gas pressure in the experiment steps were: 'before' – UHV; 'step 1' – 0.5 mbar; 'step 2' – 1 mbar; 'after' – UHV; 'reintro' – 0.5 mbar.

HT Experiment Spectra

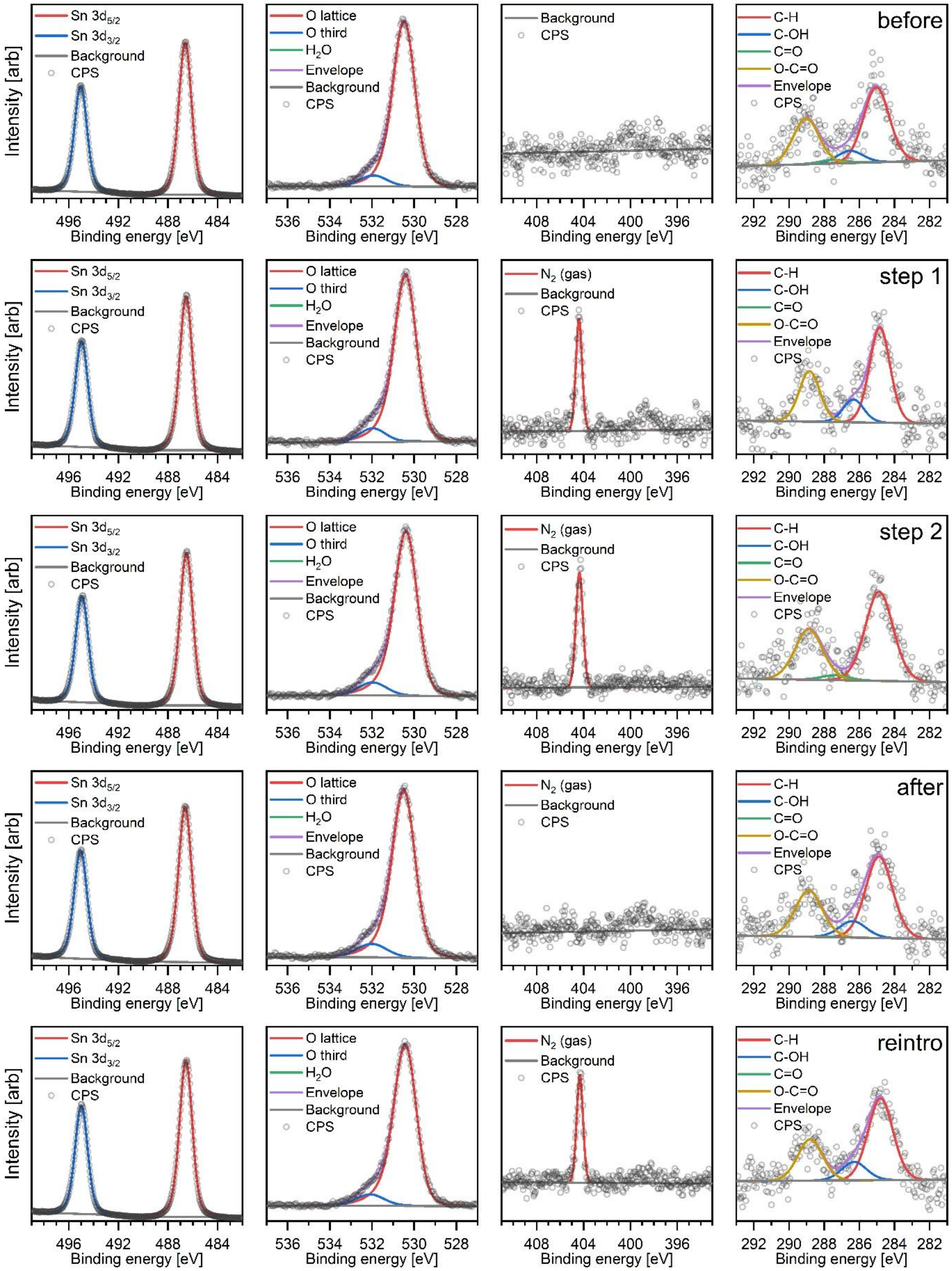

Figure S3: XP spectra of the Sn 3d, O 1s, N 1s and C 1s regions collected during the experiment HT. The label in the upper right corner of the C !s spectrum in each row corresponds to the experiment step during which that row was collected. The test gas pressure in the experiment steps were: 'before' – UHV; 'step 1' – 0.5 mbar; 'step 2' – 1 mbar; 'after' – UHV; 'reintro' – 0.5 mbar.

Materials and Sensor Preparation

This section has been reproduced from the ESI of our previous publication[1] since the exact same sensors were used in this experiment.[†]

The SnO_2 powder used in this experiment was purchased commercially (Alfa Aesar, 99.995% purity). The powder's identity was confirmed by PXRD, which gave the refined lattice parameters of a = (4.738±0.001) Å and c = (3.189±0.002) Å are in very close agreement with the literature values of a = 4.737 Å and c = 3.186 Å,[2] confirming the tetragonal, rutile-like cassiterite structure typical for SnO_2.

The sensor substrates, prepared by laser-scribing an alumina tile (0.38 mm thickness, 99.6%, Laser Cutting Ceramics Ltd) into 8x8 mm squares, were coated with 1 μm of Au (99.99%, Kurt J. Lesker) by e-beam evaporation (at 30 °C and 2.5×10^{-6} mbar base pressure, deposition rate 2.6 Å s^{-1}). Subsequently, the electrodes were etched via photolithography, using an S1818 positive photoresist and light exposure of 130 mJ cm^{-2}, followed by etching for 3600 s, using MF319 as a developer. The interdigitated electrodes produced in this way, as shown in Figure S4, had a separation between the digits of 25 μm.

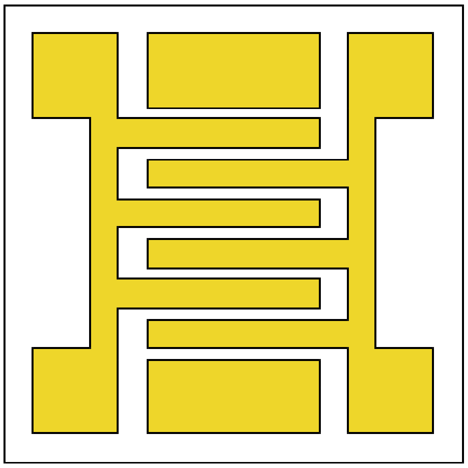

Figure S4: Layout of the electrodes on an alumina sensor platform. The electrodes are drawn to scale, with the sides of the substrate being 8 mm and the separation between the electrodes' digits being 25 μm (in the vertical direction on this diagram). The insulated Au pads were added as contact pads for the thermocouple to facilitate heat distribution and ensure accurate temperature measurements.

The sensitive layer was prepared by drop-casting an SnO_2 suspension onto the sensor substrates. The suspension was prepared from SnO_2 powder (Alfa Aesar, 99.995%

[†] DOI:10.1039/D2SC01738E, ESI: https://www.rsc.org/suppdata/d2/sc/d2sc01738e/d2sc01738e1.pdf

purity) ground with EtOH (Fisher, absolute purity) and Polyethylene Glycol 600 (Sigma Aldrich). Subsequently, the sensors were calcined in 30 ml min^{-1} O_2 flow (N5.5 purity, BOC) at 800 °C for 5 days, cooled under the same atmosphere to below 50 °C and sealed hermetically until insertion into the load lock of the spectrometer.

O calc estimation from C 1s spectra

The C 1s region was fitted with four Gaussian-Lorentzian (GL) components corresponding to hydrocarbons (C-H), alcohols (C-OH), ketones (C=O) and esters (O-C=O). The FWHM of C-OH, C=O and O-C=O were constrained to be the same as the FWHM of the C-H peak, and their BE position was constrained to, respectively, 1.5, 2.5 and 4.0 eV above the C-H peak. Following RSF normalisation of the peak intensities, the atomic ratios of 0.75, 1 and 1.5, as proposed by McIntyre et al.,[3] were assigned to C-OH, C=O and O-C=O, respectively, to estimate the total area expected in the O 1s region to be attributable to oxygen bound to carbon, i.e., originating from the carbonaceous surface contamination, which accounts for part of the O third peak found in the spectra. The full accounting of the calculated O calc values is presented in the next section.

Additional two parameters can be useful in interpreting the O calc parameter in this experiment, O calc/O third, which represents the relative involvement of organic oxygen in the observed O third peak, and O calc/O total (with O total = O lattice + O third), to estimate how much of the total detected oxygen originates from the organic contaminants, or in other words, how clean is the sample. These two parameter, presented in the next section, show that the oxygen of organic origin constitutes between 16% and 80 % of the O third peak, which means that there must be other oxygen containing species present on the surface, which could be oxygen adsorbates or hydroxyls, and that the surface is reasonably clean, with only up to a few per cent of the detected oxygen atoms in the O 1s region are likely originating from the carbonaceous contaminants, meaning the contamination is not much larger than the generally accepted detection limit in XPS of 1%.

Summary of the quantitative analysis – O/Sn, O third/Sn and O calc/Sn

Table 1: Summary of the quantitative analysis performed at each step of the experiments RT and HT, as well as the 'as-received' spectrum collected on an untreated sensor. The three values on the left hand side are referenced in the manuscript, the two values on the right are described in the preceding section.

		O/Sn	*O third/Sn*	*O calc/Sn*	*O calc/O third*	*O calc/O total*
	as received	1.29	0.47	0.10	0.21	0.06
RT	UHV before	1.23	0.09	0.04	0.49	0.03
	Step 1: 0.5 mbar	1.30	0.13	0.03	0.22	0.02
	Step 2: 1 mbar	1.33	0.19	0.03	0.16	0.02
	UHV after	1.34	0.16	0.04	0.25	0.03
	Reintro: 0.5 mbar	1.39	0.15	0.04	0.32	0.03
HT	UHV before	1.35	0.10	0.08	0.73	0.05
	Step 1: 0.5 mbar	1.39	0.13	0.09	0.71	0.06
	Step 2: 1 mbar	1.40	0.14	0.11	0.78	0.07
	UHV after	1.36	0.13	0.10	0.80	0.07
	Reintro: 0.5 mbar	1.39	0.12	0.09	0.79	0.06

References:

(1) Kucharski, S.; Ferrer, P.; Venturini, F.; Held, G.; Walton, A. S.; Byrne, C.; Covington, J. A.; Ayyala, S. K.; Beale, A. M.; Blackman, C. Direct in Situ Spectroscopic Evidence of the Crucial Role Played by Surface Oxygen Vacancies in the O_2-Sensing Mechanism of SnO_2. *Chem Sci* **2022**, *13* (20), 6089–6097. https://doi.org/10.1039/D2SC01738E.

(2) Bolzan, A. A.; Fong, C.; Kennedy, B. J.; Howard, C. J. Structural Studies of Rutile-Type Metal Dioxides. *Acta Crystallogr B* **1997**, *53* (3), 373–380. https://doi.org/10.1107/S0108768197001468.

(3) Payne, B. P.; Biesinger, M. C.; McIntyre, N. S. X-Ray Photoelectron Spectroscopy Studies of Reactions on Chromium Metal and Chromium Oxide Surfaces. *J Electron Spectros Relat Phenomena* **2011**, *184* (1–2), 29–37. https://doi.org/10.1016/j.elspec.2010.12.001.
